# Editorial: Infants with cholestasis

**DOI:** 10.3389/fped.2023.1175231

**Published:** 2023-03-28

**Authors:** Hiroo Uchida, Gregory M. Tiao, Pranavkumar Shivakumar, Kenneth K. Y. Wong, Akihiro Asai, Hizuru Amano

**Affiliations:** ^1^ Department of Pediatric Surgery, Nagoya University Graduate School of Medicine, Nagoya, Japan; ^2^ Division of Pediatric General and Thoracic Surgery, Cincinnati Children's, Hospital Medical Center, Cincinnati, OH, United States; ^3^ Division of Gastroenterology, Hepatology and Nutrition, Cincinnati Children's Hospital Medical Center, Cincinnati, OH, United States; ^4^ Department of Surgery, The University of Hong Kong, Queen Mary Hospital, Pokfulam, Hong Kong

**Keywords:** cholestasis, infant, biliary atresia, alagille syndrom, citrin deficiency, liver fibrosis

**Editorial on the Research Topic** Infants with cholestasis

Neonatal physiological jaundice is a common clinical finding that resolves spontaneously within the first 3 weeks of life. However, prolonged jaundice beyond the age of 2–3 weeks is abnormal. These infants should always have fractionated serum bilirubin levels checked to differentiate the conjugated hyperbilirubinemia of cholestasis from unconjugated hyperbilirubinemia that is usually benign and spontaneously resolves. There are numerous causes of cholestasis due to conjugated bilirubin in neonates and infants. If left untreated, cholestasis results in chronic hepatic dysfunction, which can lead to liver transplantation and even death. Therefore, it is essential to rapidly identify treatable cholestasis, such as biliary atresia or congenital biliary dilatation. Comprehensive expertise is required for early detection of cholestasis, prompt diagnostic evaluation, and adequate treatment. However, the etiology of neonatal cholestasis varies, and its incidence is low; thus, evidence-based strategies have not been fully established for cholestatic diseases. Pediatric surgeons need to update themselves with recent advances in both diagnostic and treatment strategies for cholestasis in infancy.

This special issue brings together articles that updated the diagnosis and treatment of conjugated hyperbilirubinemia in neonates and infants. Differential diagnoses of cholestasis include biliary atresia (BA) must be done. BA is an idiopathic obliterative cholangiopathy with pale stools. The incidence of BA is about 1:10,000, more commonly in Asia. The etiology of BA is still unclear. Good outcomes for BA patients depend on timely Kasai portoenterostomy. Alagille syndrome is an autosomal dominant disorder characterized by paucity of the interlobular bile ducts. The incidence is reported to be 1 in 100,000 births. Clinical features span multiple organ systems including hepatic, cardiac, vascular, renal, skeletal, craniofacial, and ocular, and occur with variable phenotypic penetrance. A majority of patients have pathogenic mutations in either the JAG 1 gene that has been mapped to chromosome 20p12, encoding a ligand for the Notch signaling pathway, or the receptor NOTCH2. Han et al. described patients with Alagille syndrome carrying novel JAG1 gene mutations. The following is a list of infantile cholestasis and the causal genes: Citrin deficiency, *SLC25A13*; Dubin-Johnson syndrome, *MRP2*; neonatal sclerosing cholangitis, doublecortin domain-containing protein 2 (DCD2) gene; PFIC1, *ATP8B1* encoding FIC1 protein; PFIC2, *ABCB11* encoding BSEP protein, a bile acid transporter; PFIC3, *ABCB4* encoding MDR3; PFIC4, *TJP2* encoding the tight junction protein TJP2; and PFIC5, *NR1H4* encoding the nuclear transcription factor FXR. Wang et al. reported three novel variants on *SLC25A13* in infants with neonatal intrahepatic cholestasis caused by citrin deficiency. Wei et al. reported novel mutations in DCDC2 in children with neonatal sclerosing cholangitis. The accuracy and cost benefit of genetic diagnosis are remarkable, and it is expected that a more accurate and rapid diagnosis will become possible in the near future, enabling rapid rule-out of biliary atresia without intraoperative cholangiography. We hope that these case reports will deepen our knowledge of these genetic diseases.

Currently, only a limited number of cases of biliary atresia do not require cholangiography for early diagnosis. MMP-7 has high diagnostic value as a preoperative marker, but it is difficult to make a definitive diagnosis (1–3). Intraoperative cholangiography, hepatic subcapsular spider-like telangiectasia, and indocyanine green fluorescence cholangiography, either alone or in combination, have been recognized for their diagnostic value, allowing for a more accurate and reliable intraoperative diagnosis of biliary atresia (4, 5).

Kasai portoenterostomy for biliary atresia early in life improves native liver survival. Less preoperative liver damage and fibrosis may lead to a better postoperative prognosis. Duan et al. described that ultrasound elastography of the liver accurately revealed liver fibrosis and was associated with the presence of postoperative esophageal varices due to portal hypertension. However, many patients are diagnosed and undergo surgery after the age of 2 months, and the prognosis of these patients is interesting, with a 50% survival rate of native liver over 10 years after surgery at 70 days of age or older by Liu et al. Although Sugita et al. reported that obstructive jaundice associated with congenital biliary dilatation could progress to chronic hepatitis and cirrhosis, radical surgery at an appropriate time could cure cirrhosis when obstructive jaundice was completely resolved. Liver fibrosis in biliary atresia also reportedly improves with enhanced bile excretion, but because biliary atresia is associated with intrahepatic bile duct abnormality, normal bile excretion is unlikely to be achieved in many cases. Sun et al. identified the transcription factors associated with postoperative bile excretion and liver fibrosis in biliary atresia, and new factors are being identified. Further studies should be conducted to confirm the functions of these factors.

The correlation among liver fibrosis, postoperative course, and portal hypertension symptoms, including these above factors, will provide new insights into the management of biliary atresia in the future.

The diagnosis of cholestasis in neonates and infants, including genetic diagnosis, has advanced considerably. Hopefully, breakthroughs will be made in the treatment of biliary atresia in the future.
